# Relationship between body mass index and masticatory factors evaluated with a wearable device

**DOI:** 10.1038/s41598-022-08084-5

**Published:** 2022-03-08

**Authors:** Shogo Yoshimura, Kazuhiro Hori, Fumiko Uehara, Shoko Hori, Yoshio Yamaga, Yoko Hasegawa, Kohei Akazawa, Takahiro Ono

**Affiliations:** 1 Division of Comprehensive Prosthodontics, Faculty of Dentistry and Graduate School of Medical and Dental Sciences, Niigata University, 2-5274 Gakkocho-dori, Niigata, 951-8514 Japan; 2 Department of Medical Informatics, Niigata University Medical and Dental Hospital, Niigata, 951-8520 Japan

**Keywords:** Health care, Dentistry, Nutrition and diet in dentistry

## Abstract

Numerous studies have evaluated the relationship between eating behavior and obesity, however few studies have objectively assessed eating behavior. Additionally, the association of masticatory behaviors with masticatory performance remains unclear. This study aimed to verify the relationship between masticatory performance and behavior measured by a wearable masticatory counter, and BMI. 365 healthy adults participated. Mastication behaviors, i.e. number of chews and bites, chewing rate, and chewing time, were measured using wearable masticatory counter while consuming one rice ball (100 g). Masticatory performance was evaluated using testing gummy jelly. Lifestyle habits including exercise, walking, and breakfast, were surveyed by questionnaire. The correlation coefficients between masticatory behaviors and performance and BMI were analyzed. Furthermore, multiple regression analysis was performed. The number of chews showed positive correlation with chewing rate, number of bites and chewing time, but no correlation with masticatory performance. BMI had weak but significant negative correlation with number of chews, bites, chewing time, and masticatory performance, but had no correlation with chewing rate. Multiple regression analysis revealed that BMI was associated with sex, age, number of chews, bites, masticatory performance, and walking speed. In conclusion, masticatory behavior and performance were not interrelated, but both were independently associated with BMI weakly.

## Introduction

In recent years, the global population of individuals with obesity has continued to increase due to changes in eating habits, diversification of diets such as fast food, and lack of exercise^1,2^. Obesity is not just overweight, and it is a condition involving excessive accumulation of body fat, which has been reported to affect various systemic diseases such as diabetes, dyslipidemia, hypertension, and cardiovascular disease^3–6^. Previous studies have identified the involvement of other factors, including lack of exercise, smoking, excessive drinking, excessive stress, eating alone, occupation, parents’ education, short sleep duration, and lack of breakfast^7–11^. Many studies using subjective data have also examined the relationship between eating behavior and obesity, reporting that eating behaviors such as “eating quickly” and “lower chewing frequency” lead to obesity. In one study of a large adult Japanese population, Otsuka et al.^12^ investigated the association between eating quickly and BMI at the time of the survey, and changes in BMI from that at 20 years of age. They reported that participants who ate quickly had higher BMI and a greater BMI increase since 20 years of age. In addition, Fukuda et al.^13^ examined the relationship between the number of chews and weight gain of more than 10 kg since the age of 20 years in middle-aged adults, and reported that participants with a lower chewing frequency were at a 9.1-times higher risk of weight gain.

However, many of these previous studies were based on subjective data from self-administered questionnaires, or objective data measured in unusual abnormal situations, such as experiments using large jaw movement measurement devices^14^, electromyograms^15^, and video recordings of meals ^16^. Because no reports have objectively measured mastication in usual meal situations, it remains unclear which mastication factors (e.g., the number of chews and the chewing rate) are related to BMI.

Sharp Co. (Sakai, Japan) developed a small ear-hung masticatory counter device, called Bitescan, for measuring masticatory behavior in usual situations^17^. This wearable masticatory counter is designed to assess objective masticatory behavior just by putting on the ear. We had confirmed the validity of this mastication counter^17^.

Some previous studies reported that a decrease in masticatory performance, which indicates the quality of mastication, is a risk factor for lifestyle-related diseases, such as metabolic syndrome^18–20^. Kikui et al.^18^ reported that individuals with low masticatory performance had a 1.46-times higher risk of developing metabolic syndrome than the group with high masticatory performance. Other studies^19,20^ investigating the relationship between masticatory performance and diet reported that masticatory performance was closely related to the type of meal an individual usually ate. Regarding the relationship between the hardness of foods and energy intake, Bolhuis et al.^21^ reported that energy intake was increased when a soft meal was eaten, compared with a hard meal. Therefore, since masticatory performance contributes to energy intake, it may be an important factor in the relationship between mastication and obesity.

Based on these back grounds, we hypothesized that the mastication behavior measured objectively had the relationship between BMI. Furthermore, clarifying the relationship between masticatory performance and masticatory behavior would be important for understanding the relationship between obesity and mastication. The current study aimed to verify the relationship between masticatory performance and behaviors when a specified amount of food was ingested, and to verify the association between BMI and masticatory behaviors and performance.

## Materials and methods

### Participants

This study was designed as an exploratory study to investigate the relationship between BMI and mastication. 365 healthy adults (203 men and 162 women, average age 36.6 ± 12.1 years) participated in this study. Inclusion criteria were used to select healthy adult volunteers aged 20 to 70 years who understood the purpose of the study. We excluded participants who had eating disorders, dysphagia, subjective or objective abnormalities in temporomandibular joint/stomatognathic function, dental pain, periodontal problems, those who were undergoing dental treatment or orthodontic treatment, and those taking medication for diabetes or hyperlipidemia. After the study purpose was explained, all participants provided written informed consent. This study was approved by the Institutional Review Board of Niigata University (approval number 2017-0230). All procedures performed in this study were in accordance with the ethical standards of the institutional and national guideline committee and with the 1964 Helsinki declaration and its later amendments or comparable ethical standards.

## Measurement items

### Masticatory behavior

**Figure 1:**
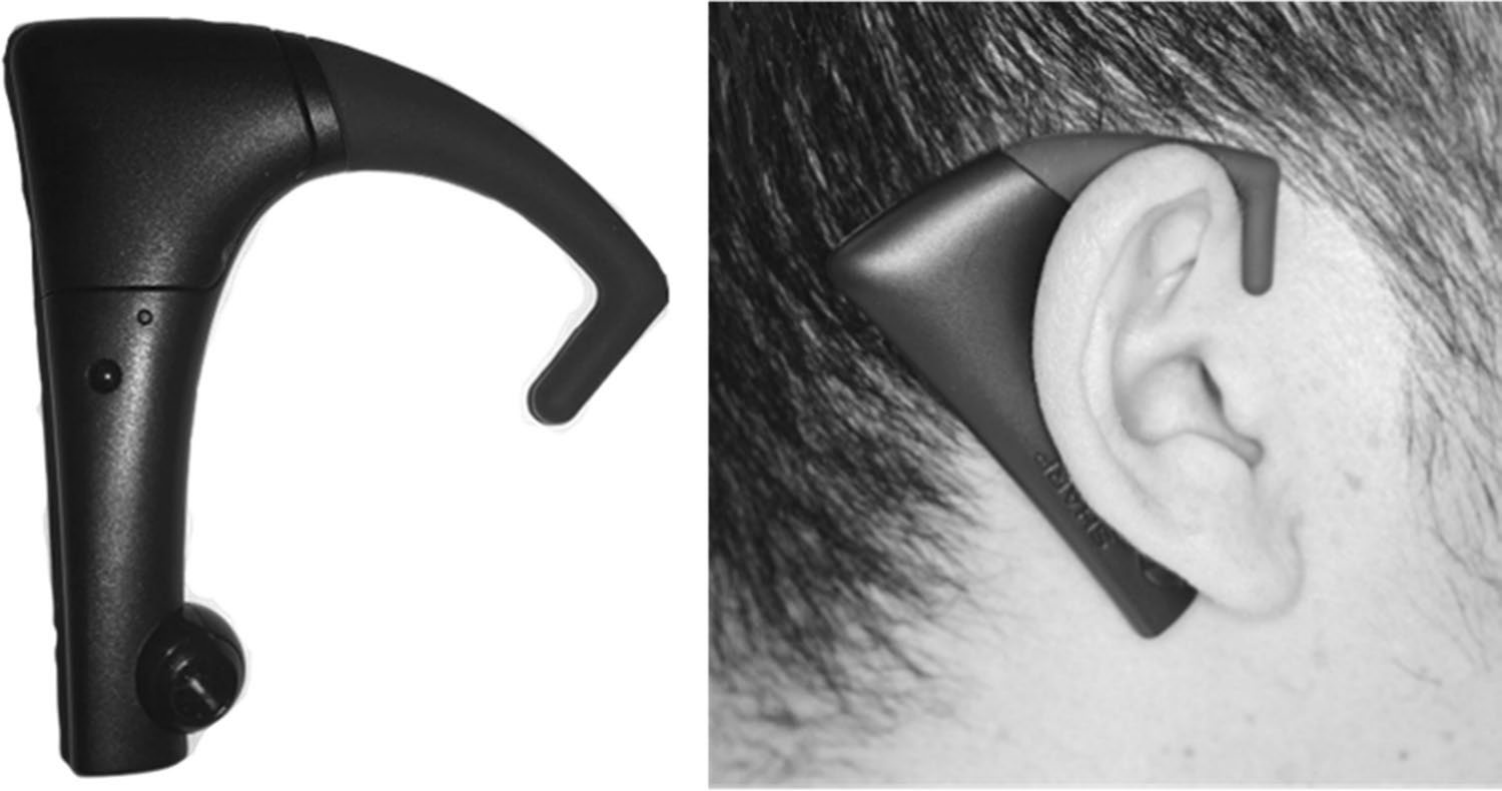
The masticatory counter (Bitescan).

Masticatory behavior was measured using Bitescan (Fig. 1, Sharp Co., Sakai, Japan)^17^. This wearable device has an infrared distance sensor and scans morphological changes in the skin surface on the posterior side of the right pinna by mastication at 20 Hz. Three different ear-hook sizes (S, M, L) were prepared, so we could adjust the device and use the ear-hook best suited to each participant’s pinna. Before measurement, we fitted the device to ensure that the sensor was correctly located on the back of participants’ pinna. The device was connected to a smartphone (SHM05, Sharp Co., Sakai, Japan) via Bluetooth and data were collected with a smartphone application.

After the appropriately sized Bitescan ear-hook was selected, participants were asked to consume one rice ball (100 g, seaweed-rolled rice balls, Marusan, Higashi-Osaka, Japan) freely with no specific instructions except: “Please eat one rice ball as usual”. Rice balls, formed by cooked rice and wrapped in seaweed, are familiar to Japanese people, and are typically eaten by hand rather than using chopsticks, spoons, or forks.

Data were analyzed using a smartphone application and dedicated software. In this study, masticatory behavior was measured as the number of chews, chewing rate, number of bites, and total chewing time. The number of chews was defined as the total number of mastication cycles in tasks, and the chewing rate was defined as the number of chews per minute calculated by dividing by the total chewing time. The number of bites was defined as the sum of uptake actions performed until the test food was completely eaten. Total chewing time was defined as the sum of the time from the uptake of food to swallowing. The time before uptake or between swallowing and re-uptake was not included in the total chewing time.

### Masticatory performance

Masticatory performance was measured using a test gummy jelly (20 × 20 × 10 mm, 5.5 g, UHA Mikakuto Co., Ltd., Osaka, Japan) and an image analysis application for masticatory performance measurement^22^. This method was used for evaluating masticatory performance by calculating the surface area of the comminuted gummy jelly and reported the validity and reliability^23–28^. Participants were asked to chew the gummy jelly 30 times without swallowing, then expectorate the comminuted pieces onto gauze. Collected specimens were then washed with tap water to prevent further dissolution and transferred into a box containing 30 ml of water to prevent overlap of the particles. The box was a polystyrene case (inner dimensions 140 mm × 95 mm × 36 mm, Daiso, Higashihiroshima, Japan) with black markers (7 mm x 7 mm, distance between markers-width: 88 mm, length 133 mm) at the four corners. Digital images of the box containing the comminuted pieces were carefully taken including the four markers using a smartphone camera with an analysis application. The application estimated the surface area increase from the RGB information of the specimen and calculated that value as the masticatory performance.

### Anthropometric measurement

Height was measured with a height meter (seca213, seca, Chiba, Japan). BMI was calculated by the body composition analyzer (RD-503, Tanita, Tokyo, Japan). For body composition measurement, participants were instructed to wear clothing that was as light as possible, and we subtracted an estimated clothing weight of 1.0 kg.

### Self-administered questionnaire

Participants answered a self-administered questionnaire: “Do you exercise regularly for 30 minutes or more?”, “Do you walk faster than other people of the same sex/generation?”, “Do you usually have breakfast every day?". The three questions were answered “Yes” or “No”. The question “How many hours do you sleep on average?” was answered in 0.5-h units.

To assess participants’ food intake habits, the brief-type self-administered diet history questionnaire (BDHQ, Gender Medical Research, Tokyo, Japan) ^29,30^ was administered. The BDHQ is a dietary questionnaire developed for Japanese people, enabling estimation of the intake of various nutrients and foods, and the validity of this questionnaire was reported^29,31^. From the results, the amount of total energy intake, the amount of energy intake of carbohydrates, the amount of energy intake of fat, and the adjusted total dietary fiber amount (/4186kJ [1000 kcal]) were calculated.

## Statistical analysis

After examining normality, the average for each measurement item was calculated and comparison between sexes was performed using student’s t-tests. Univariate analysis was performed using Pearson’s correlation coefficients to clarify the relationship between masticatory behaviors and performance, the relationship between masticatory behaviors and energy intake, and the relationship between BMI and measurement items (except the questionnaire results). Additionally, participants were divided into two groups based on the questionnaire results (“exercise regularly?”, “walking faster?”, “eating breakfast?”). The mean BMI in each group was calculated, and masticatory behaviors were compared between the two groups using student's t-tests.

The complex associations of masticatory behaviors, masticatory performance, lifestyle, nutritional status, and BMI were analyzed using multiple regression analysis (stepwise procedure). The dependent variable was BMI, and the explanatory variables were sex, age, the number of chews, number of bites, chewing rate, total chewing time, masticatory performance, questionnaire results, energy intake, carbohydrate intake, lipid intake, and adjusted total amount of dietary fiber. Furthermore, the multiple regression analysis by sex group was also performed.

In this study, we determined the sample size for multivariate analysis for each gender. The explanatory variables in the multivariate analysis model we set were 14. According to Green's report^32^, it was calculated that 50 + 8 * 14 = 162 was required as the sample size for both men and women.

Statistical analyses were conducted using SPSS (Ver. 24.0, for Windows) (SPSS Inc., Chicago, IL, USA). The significance level was set at *p* = 0.05.

## Results

### Comparison of BMI and mastication between sexes (Table 1)

The mean BMI was 22.5 ± 3.4 (kg/m^2^), range was 13.2–37.1. According to WHO criteria, 21 (5.8%) was classified as underweight, 274 (75.1%) as normal, 56 (15.3%) as overweight, and 14 (3.8%) as obesity.

**Table 1:** Characteristics of participants.

	All (n = 365)	Men (n = 203)	Women (n = 162)	*p* value
Age (y)	36.6 ± 12.1	37.8 ± 11.6	35.0 ± 12.5	0.025
BMI (kg/m^2^)	22.5 ± 3.4	23.4 ± 3.3	21.4 ± 3.2	< 0.001
Number of chews (chews)	212.9 ± 103.3	174.4 ± 75.1	261.2 ± 113.2	< 0.001
Number of bites (bites)	10.5 ± 6.4	9.0 ± 5.4	12.5 ± 7.0	< 0.001
Chewing rate (chews/min)	78.3 ± 13.3	79.3 ± 13.4	77.1 ± 13.2	0.115
Total chewing time (sec)	162.4 ± 74.0	131.4 ± 51.0	201.1 ± 79.9	< 0.001
Masticatory performance (mm^2^)	5076.0 ± 1308.7	5164.4 ± 1253.2	4965.2 ± 1371.0	0.149
Sleep duration (h)	6.2 ± 0.9	6.1 ± 0.8	6.3 ± 0.9	0.076
Total energy intake (kJ/day)	7689.7 ± 2414.5	8424.7 ± 2452.2	6769.2 ± 2025.6	< 0.001
Fat energy intake (kJ/day)	2056.2 ± 729.2	2177.6 ± 727.5	1903.8 ± 704.1	< 0.001
Carbohydrate energy intake (kJ/day)	4032.0 ± 1424.9	4456.4 ± 1499.8	3499.5 ± 1121.0	< 0.001
Dietary fiber intake (g/4186 kJ)	6.2 ± 2.0	5.8 ± 1.7	6.6 ± 2.1	< 0.001

Student’s *t* test (comparison between men and women participants).

The values in the table indicate mean ± standard deviation.

The BMI was significantly lower in women than in men (*p* < 0.001). The average of the number of chews, number of bites, chewing rate, total chewing time, and masticatory performance in all subjects were 212.9 ± 103.3 (chews), 10.5 ± 6.4 (bites), 78.3 ± 13.3 (chews/min), 162.4 ± 74.0 (s), and 5076.0 ± 1308.7 (mm^2^), respectively.

The number of chews (*p* < 0.001), number of bites (*p* < 0.001), and total chewing time (*p* < 0.001) were significantly smaller in women, but there were no significant differences in chewing rate (*p* = 0.115) and mastication performance (*p* = 0.149) between men and women.

There was no significant difference in sleep duration between men and women (*p* = 0.076). The total energy intake (*p* < 0.001), lipid energy intake (*p* < 0.001), and carbohydrate energy intake (*p* < 0.001) were significantly higher in men than in women, but the dietary fiber intake (*p* < 0.001) was higher in women than in men.

### Relationship between masticatory behavior and performance (Table 2)

The number of chews was significantly positively correlated with the number of bites (r = 0.484), chewing rate (r = 0.367), and total chewing time (r = 0.919), However, none of parameters of masticatory behaviors were associated with masticatory performance (Table 2).

**Table 2:** Correlation of objective data.

	BMI	Number of chews	Number of bites	Chewing rate	Total chewing time	Masticatory performance
BMI		− 0.297**	− 0.252**	− 0.031	− 0.296**	− 0.150**
Age	0.250**	− 0.024	− 0.091	0.143**	− 0.064	− 0.162**
Number of chews	− 0.297**		0.484**	0.367**	0.919**	0.041
Number of bites	− 0.252**	0.484**		0.597**	0.597**	− 0.001
Chewing rate	− 0.031	0.367**	− 0.085		0.050	0.094
Total chewing time	− 0.296**	0.919**	0.597**	0.050		0.031
Masticatory performance	− 0.150**	0.041	− 0.001	0.094	0.031	
Total energy	0.137**	− 0.085	− 0.179**	0.169**	− 0.159**	0.055
Fat energy	0.043	− 0.021	− 0.117*	0.148**	− 0.077	0.090
Carbonhydrate energy	0.137**	− 0.091	− 0.159**	0.145**	− 0.158**	0.030
Dietary fiber intake	− 0.127*	0.208**	0.031	0.087	0.188**	0.040

Pearson coefficients : **p < 0.01, *p < 0.05.

### Relationship between mastication and nutrition (Table 2)

There was no significant correlation between total energy intake and number of chews (r = −0.085), and masticatory performance (r = 0.055). Total energy intake had a significant weak negative correlation with the number of bites (r = −0.179), and total chewing time (r = −0.159). In addition, there was significant weak positive correlation between total energy intake and chewing rate (r = 0.169).

Lipid energy intake had a significant weak negative correlation with number of bites (r = −0.117), and a significant weak positive correlation with chewing rate (r = 0.148).

Carbohydrate energy intake had significant weak negative correlations with number of bites (r = −0.159) and total chewing time (r = −0.158), and a significant weak positive correlation with chewing rate (r = 0.145).

Dietary fiber intake had a significant weak positive correlation with number of chews (r = 0.208) and total chewing time (r = 0.188) (Table 2).

### Relationship with BMI between mastication and nutrition (Table 2), and comparison based on the questionnaire (Table 3)

**Table 3:** Comparison between groups based on questionnaire.

		Yes		No		*p* value
		n	BMI	n	BMI	
All	Exercise regularly	101 (27.7%)	22.9 ± 3.4	264 (72.3%)	22.4 ± 3.4	0.164
	Walking faster	194 (53.2%)	22.4 ± 3.2	171 (46.8%)	22.7 ± 3.6	0.131
	Eating breakfast?	292 (80.0%)	22.6 ± 3.3	73 (20.0%)	22.3 ± 3.8	0.142
Men	Exercise regularly	62 (30.5%)	23.7 ± 3.6	141 (69.5%)	23.3 ± 3.1	0.382
	Walking faster	116 (57.1%)	23.4 ± 3.2	87 (42.9%)	23.4 ± 3.4	0.910
	Eating breakfast	159 (78.3%)	23.4 ± 3.2	44 (21.7%)	23.4 ± 3.6	0.860
Women	Exercise regularly	39 (24.1%)	21.6 ± 2.4	123 (75.9%)	21.3 ± 3.4	0.569
	Walking faster	78 (48.1%)	20.8 ± 2.6	84 (51.9%)	21.8 ± 3.5	0.038
	Eating breakfast	133 (82.1%)	21.5 ± 3.0	29 (17.9%)	20.7 ± 3.5	0.285

Student’s *t* test (comparison of BMI between Yes or No).

The values in the table indicate mean ± standard deviation.

BMI had a significant weak positive correlation with age (r = 0.250).

Additionally, BMI had significant weak negative correlations with number of chews (r = −0.297), number of bites (r = −0.252), total chewing time (r = −0.296), and masticatory performance (r = −0.150). However, there was no significant correlation with chewing rate (r = −0.031).

Regarding energy intake, BMI had significant weak positive correlations with total energy intake (r = 0.137), and carbohydrate energy intake (r = 0.137), and a weak negative correlation with dietary fiber intake (r = −0.127). There was no significant correlation between BMI and lipid energy intake (r = 0.043) (Table 2).

Comparison of the two groups based on questionnaire results revealed that the walking faster group had lower BMI than the slower group in women, with no significant differences in other items (Table 3).

### Multiple regression analysis for BMI and mastication (Tables 4 and 5)

**Table 4:** All participants—Multiple regression analysis for BMI.

Explanatory variable	Unstandardized coefficients					95% Confidence interval for B	
	B	SD	β	t	*p* value	Lower	Upper
(Constant)	24.080	0.971		24.806	< 0.001	22.171	25.989
Sex	− 1.354	0.360	− 0.199	− 3.765	0.001	− 2.062	− 0.647
Age	0.057	0.014	0.202	4.163	< 0.001	0.030	0.083
Number of chews	− 0.005	0.002	− 0.155	− 2.689	0.007	− 0.009	− 0.001
Masticatory performance	0.000	0.000	− 0.125	− 2.597	0.010	− 0.001	0.000
Walking faster	0.678	0.322	0.100	2.102	0.036	0.044	1.312
Number of bites	− 0.059	0.029	− 0.112	− 2.062	0.040	− 0.116	− 0.003

Explanatory variables: Sex, Age, Number of chews, Number of bites, Chewing rate, Total chewing time, Masticatory performance, Total energy intake, Fat energy intake, Carbonhydrate energy intake, Dietary fiber intake, Sleep duration, Exercise, Walking, Breakfast.

Stepwise procedure: *p* < 0.05, Adjusted R^2^ = 0.194.

**Table 5:** Grouped by sex—Multiple regression analysis for BMI.

	Explanatory variable	Unstandardized coefficients					95% Confidence Interval for B	
		B	SD	β	t	*p* value	Lower	Upper
Men	(Constant)	23.103	0.920		25.102	< 0.001	21.288	24.918
	Number of chews	− 0.009	0.003	− 0.211	− 3.110	0.002	− 0.015	− 0.003
	Age	0.051	0.019	0.180	2.653	0.009	0.013	0.089
Women	(Constant)	19.598	0.911		21.522	< 0.001	17.799	21.397
	Age	0.077	0.019	0.304	4.134	< 0.001	0.040	0.114
	Number of chews	− 0.006	0.002	− 0.200	− 2.718	0.007	− 0.010	− 0.002
	Walking faster	1.061	0.465	0.168	2.279	0.024	0.141	1.980

Explanatory variables: Age, Number of chews, Number of bites, Chewing rate, Total Chewing time, Masticatory performance, Total energy intake, Fat energy intake, Carbonhydrate energy intake, Dietary fiber intake, Sleep duration, Exercise, Walking, Breakfast.

Men : Stepwise procedure: *p* < 0.05, Adjusted R^2^ = 0.068.

Women : Stepwise procedure: *p* < 0.05, Adjusted R^2^ = 0.137.

Multiple regression analysis revealed that BMI was negatively associated with sex, number of chews, number of bites, masticatory performance, and walking speed, and was positively associated with age. Thus, older age, being men, fewer chews, fewer bites, lower masticatory performance, and slow walking speed were associated with higher BMI (Table 4).

According to the results by sex, older age and fewer chews were associated with higher BMI in men, and slower walking speed was associated with BMI in women (Table 5).

There was no multicollinearity in these models.

## Discussion

In the current study, we subdivided mastication into components such as masticatory behaviors and performance, and examined the relationship between masticatory behaviors and performance. The results revealed that masticatory behaviors and performance had no associations or interactions with each other. Furthermore, it was revealed that the elements of mastication (behavior and performance) were independently associated with BMI weakly, even when various factors were taken into consideration. As expected, the current method revealed the relationship between mastication and BMI in more detail compared with conventional subjective evaluation, with several methodological advantages.

Most previous studies measuring masticatory behavior and jaw movement objectively utilized special equipment, such as attaching electromyographic electrodes to measure the masseter and temporal muscle activity^15^, attaching sensors or magnets to the mandibular anterior tooth to measure jaw movement^14^, and using video recordings of a meal scene to track jaw movements^16^. These methods can accurately measure masticatory movement, but involve situations that differ from usual daily eating behavior and they were sometimes wired devices. Bitescan can measure masticatory behavior when attached to the ear without restraint, and can conduct measurement without disturbing usual eating behavior. Although many previous studies of masticatory behavior have examined around 10 participants due to measurement limitations^15^, we were able to examine 365 healthy adults because of the lightweight and convenient characteristics of Bitescan device. In our previous study, the accuracy of Bitescan for masticatory behaviors was found to be equivalent to that of a conventional jaw movement measuring device, and measurement with Bitescan was predicted to be useful for elucidating mastication^17^.

The rate of obesity in Japanese was higher in males (according to The National Health and Nutrition Survey in Japan^33^), and in fact, even in this study, males had a significantly higher BMI. Therefore, we analyzed masticatory behavior and investigated factors related to BMI by sex. The number of chews, number of bites, and total chewing time were significantly larger in women than in men, consistent with findings reported by Park et al.^34^. The finding of no significant difference between men and women in chewing rate was also consistent with Tamura et al.’s findings^35^.

Masticatory performance had no significant associations with the number of chews, number of bites, chewing rate, or total chewing time. We initially expected that people with low masticatory performance would exhibit decreased food biting, crushing, and mixing ability in the process of bolus formation, and that masticatory behavior would be modulated to compensate for decreased function. However, our results did not indicate that compensatory modulation occurred. This lack of correlation between the number of chews and masticatory performance might have occurred because the participants in this study were healthy adults in their 20s and 50s. Additionally, most participants did not have compromised dentition. The mean number of residual teeth of participants was 28.1 ± 2.3 (median 28) and they had sufficient occlusal support. The exclusion criteria we set excluded participants with oral hypofunction. Therefore, it was unlikely that masticatory ability would decline due to aging or missing teeth. In addition, we did not include the number of remaining teeth and the number of occlusal supports as factors. If older participants and/or individuals whose masticatory performance was significantly reduced due to tooth loss were included, a correlation between the number of chews and masticatory performance might be present, and compensatory modulation might occur. To the best of our knowledge, masticatory behavior and performance are both important factors in mastication, but no previous studies have investigated their association. The results of the current study extend current knowledge of the relationship between masticatory behavior and performance.

We investigated the effects of lifestyle factors related to obesity, which have been reported in many studies^7–11^, but only walking speed was significant in the multivariate analysis. Associations between BMI and overweight status, walking speed, and regular exercise have been reported in older people^36,37^, and exercise therapy for patients with diabetes and/or metabolic syndrome has been shown to be effective^38^. The multiple regression analysis results that faster walking speed, compared with the same age group in women, was associated with lower BMI could have been caused by an increase in basal exercise and an improvement in basal metabolism.

Cappuccio et al.^39^ reported that adults with short sleep duration of less than 5 h were significantly more likely to become obese than those with longer sleep duration. Short sleep duration is associated with decreased leptin secretion, which stimulates the satiety center produced by adipocytes, and increases secretion of ghrelin, which is produced in the stomach and promotes eating^40^. It has been reported that ghrelin not only enhances eating, but also improves eating speed and appetite for oily foods^41^. In our study, the overall average sleep duration was 6.2 ± 0.9 h, and few subjects had short sleep duration. In future studies, if the sample size is increased and divided into obese and non-obese groups, similar results to Cappuccio's would be expected^39^.

Our results revealed that BMI was weakly but significantly negatively associated with the number of chews, number of bites, and masticatory performance. An increase in the number of chews generally increases the plasma concentration of gastrointestinal hormones such as insulin, GLP-1 (Glucagon-like peptide), ghrelin, and CCK (Cholecystokinin), and leads to an improvement in fullness, suppression of postprandial blood glucose, and enhancement of digestive ability^42–44^. In addition, an association was reported between an increased number of chews and reduced food intake and energy intake^45^. A large number of bites with the prescribed amount of food, as in this study, indicates that the amount of intake per bite was small. Sun et al.^46^ reported that reducing the amount of intake per bite reduced the number of chews per bite, but the number of bites also increased; thus, the total number of chews increased and blood glucose levels rose slowly. Ingesting food in smaller amounts would be expected to indirectly lead to an increased number of chews, more hormone secretion, and a feeling of fullness. Thus, weight gain is suppressed by not ingesting more food than necessary; hence, BMI values may be lower. Based on our results, the masticatory behaviors associated to low BMI were consistent with a previous report comparing the masticatory behaviors of obese and underweight people^47^.

Although some previous studies reported that “eating quickly” leads to obesity^12^, other studies reported no association^48^. The inconsistency in results may be due to ambiguity in the definition of “eating quickly”. In the current study, the masticatory behaviors associated with BMI were the number of chews, number of bites, and total chewing time; chewing rate showed no association. Accordingly, the findings suggested that the eating behavior as “eating quickly” which the number of bites was small (a large amount ingested in each bite) and a small number of chews per food weight indicated short total chewing time.

Some previous studies reported that particles of the pre-swallowing bolus are larger in obese and underweight individuals compared with healthy individuals, and that reduced masticatory performance might shift the eating preference to soft foods rich in carbohydrates and fats^49,50^. Because most participants in our study were healthy adults without tooth loss, a preference for soft food because of difficulty chewing was unlikely. The current findings revealed no association between masticatory performance and energy intake of carbohydrates and lipids. N’gom et al.^51^ reported that, when mastication was inadequate, food was excreted whole, and indigestion occurred. Farrell^52^ reported that beef tallow and rice were easily digested without chewing, whereas fibrous vegetables and roasted meat were not. Easily absorbed foods such as fat and carbohydrates are absorbed well, even with low masticatory performance, but some nutrients, such as vitamins and dietary fiber, are excreted from the body because of indigestion. These phenomena may be related to high BMI values in people with reduced masticatory performance. To clarify the relationship between masticatory performance and BMI in healthy adults with few tooth defects, the relationships between the properties of the bolus, nutrient absorption, and gastrointestinal hormones, which are considered to be affected by masticatory performance, should be examined in the future.

The energy intake calculated by the BDHQ was negatively correlated with the number of bites and total chewing time, but was not related to the number of chews. In this study, in which the prescribed amount of a 100 g rice ball was ingested, no association was found between daily energy intake and the number of chews, because the same of amount of food was ingested. The finding that the number of bites was small and the total chewing time was short suggests that the usual amount of each bite was large, in accord with the multiple regression analysis. If the number of chews per amount of food intake was measured, the relationship between energy intake and the number of chews might be clarified.

Because dietary fiber intake was positively correlated with the number of chews and total chewing time, but unrelated to the number of bites, the results indicated that people who consumed more dietary fiber performed specific behaviors, such as frequent chewing taking more time. It is possible that individuals who regularly prefer to consume high-fiber foods were already accustomed to behavioral patterns that did not involve “eating quickly”.

In our study, both the lifestyle and the energy intake data were based on self-administered questionnaires. Since these questionnaires had not been verified, these subjective data may have decreased the accuracy of the analysis. Increasing accuracy by measuring actual walking speed, surveying intensity of regular exercise and breakfast skipping rate may enable detailed investigation of the strength of these relationships. Dietary intake is an important factor in obesity and eating behavior. In our study, the energy intake by BDHQ and each nutrient could be significantly but considerably weakly correlated with BMI. BDHQ^53,54^ and other dietary history survey^55^ forms reported the possibility of underreporting or overreporting. Especially in BDHQ, there was a report that a high degree of obesity was likely to be underreported^53,54^. However, considering the burden on participants, it might be difficult to record the contents of daily meals one by one. Future studies could verify the relationship between mastication and actual daily intake by diet report and/or monitoring daily meals using Bitescan device, which can add a meal image recognition and, instead of BDHQ. As a basic study, we prepared rice balls (100 g), so that food intake was easy to regulate, and conducted an experiment. Rice balls are familiar to Japanese people, and are typically eaten by hand rather than using chopsticks, spoons, or forks. Therefore, the bite amount is easily adjusted, possibly affecting the size of bites. Eating utensils may affect bite size.

Despite these limitations, the current study is the first to report that masticatory behavior and performance are independently associated with BMI weakly. The results revealed that low BMI was associated with a small amount of intake (a large number of bites), a large number of chews, and higher masticatory performance. Although this was a cross-sectional study, for future studies to clarify the longitudinal relationship between masticatory behavior and BMI, it will be necessary to examine masticatory behavior change using an intervention test. Because our device uses a smartphone application and is relatively small in size, we predict that it could be easily incorporated into daily life to change eating behavior. In addition, to improve masticatory performance, appropriate dental treatment and training could be performed to increase occlusal force. In future, we plan to investigate the prevention and improvement of obesity by changing mastication.

## Conclusions

Masticatory behavior and performance were not correlated. However, the number of chews, number of bites, and masticatory performance had weak association with BMI independently.
